# Two‐Year Outcomes With a Next‐Generation Left Atrial Appendage Device: Final Results of the PINNACLE FLX Trial

**DOI:** 10.1161/JAHA.122.026295

**Published:** 2023-02-15

**Authors:** Shephal K. Doshi, Saibal Kar, Ashish Sadhu, Rodney Horton, Jose Osorio, Christopher Ellis, James Stone, Manish Shah, Srinivas R. Dukkipati, Stuart Adler, Devi G. Nair, Jamie Kim, Oussama Wazni, Matthew J. Price, David R. Holmes, Robert Shipley, Thomas Christen, Dominic J. Allocco, Vivek Y. Reddy, the PINNACLE FLX investigators, Stuart Adler, Maurice Buchbinder, Larry Chinitz, David Delurgio, Amish Desai, Health Center, Shephal Doshi, Srinivas R. Dukkipatti, Christopher Ellis, Douglas Gibson, David Holmes, Rodney Horton, Kenneth Huber, Saibal Kar, Farhat Khairallah, Jamie Kim, Jayanthi Koneru, Paul Mahoney, Moussa Mansour, George Mark, Devi Nair, William O’Neill, William Nicholson, Jose Osorio, Matthew J. Price, Vivek Y. Reddy, James Reiss, Ashish Sadhu, Walid Saliba, Manish Shah, Michael Shehata, James Stone, Vijendra Swarup

**Affiliations:** ^1^ Providence, Saint Johns Health Center Pacific Heart Institute Santa Monica CA; ^2^ Los Robles Regional Medical Center Thousand Oaks CA; ^3^ Phoenix Cardiovascular Research Group Phoenix AZ; ^4^ Texas Cardiac Arrhythmia Austin TX; ^5^ Arrhythmia Institute at Grandview Birmingham AL; ^6^ Vanderbilt University Medical Center Nashville TN; ^7^ North Mississippi Medical Center Tupelo MS; ^8^ MedStar Health Medical Center Washington DC; ^9^ Helmsley Electrophysiology Center, Icahn School of Medicine at Mount Sinai New York NY; ^10^ M Health Fairview East Region St Paul MN; ^11^ St Bernard’s Heart and Vascular Center Jonesboro AR; ^12^ New England Heart and Vascular Institute at Catholic Medical Center Manchester NH; ^13^ Cleveland Clinic Cleveland OH; ^14^ Scripps Clinic La Jolla CA; ^15^ Mayo Clinic Rochester MN; ^16^ Boston Scientific Corporation Marlborough MA

**Keywords:** atrial fibrillation, left atrial appendage closure, WATCHMAN FLX, Atrial Fibrillation, Ischemic Stroke, Catheter-Based Coronary and Valvular Interventions

## Abstract

**Background:**

The PINNACLE FLX (Protection Against Embolism for Non‐valvular AF [Atrial Fibrillation] Patients: Investigational Device Evaluation of the Watchman FLX LAA [Left Atrial Appendage] Closure Technology) trial evaluated the safety and efficacy of a next‐generation left atrial appendage closure device (WATCHMAN FLX; Boston Scientific, Marlborough, MA). At 1 year, the study met the primary end points of safety and anatomical efficacy/appendage closure. This final report of the PINNACLE FLX trial includes the prespecified secondary end point of ischemic stroke or systemic embolism at 2 years, also making it the first report of 2‐year outcomes with this next‐generation left atrial appendage closure device.

**Methods and Results:**

Patients with nonvalvular atrial fibrillation with CHA_2_DS_2_‐VASc score ≥2 (men) or ≥3 (women), with an appropriate rationale for left atrial appendage closure, were enrolled to receive the left atrial appendage closure device at 29 US centers. Adverse events were assessed by an independent clinical events committee, and imaging was assessed by independent core laboratories. Among 395 implanted patients (36% women; mean age, 74 years; CHA_2_DS_2_‐VASc, 4.2±1.5), the secondary efficacy end point of 2‐year ischemic stroke or systemic embolism was met, with an absolute rate of 3.4% (annualized rate, 1.7%) and an upper 1‐sided 95% confidence bound of 5.3%, which was superior to the 8.7% performance goal. Two‐year rates of adverse events were as follows: 9.3% all‐cause mortality, 5.5% cardiovascular death, 3.4% all stroke, and 10.1% major bleeding (Bleeding Academic Research Consortium 3 or 5). There were no additional systemic embolisms, device embolizations, pericardial effusions, or symptomatic device‐related thrombi after 1 year.

**Conclusions:**

The secondary end point of 2‐year stroke or systemic embolism was met at 3.4%. In these final results of the PINNACLE FLX trial, the next‐generation WATCHMAN FLX device demonstrated favorable safety and efficacy outcomes.

## Registration

URL: https://www.clinicaltrials.gov; Unique identifier: NCT02702271.

Transcatheter left atrial appendage closure (LAAC) is recommended as a nonpharmacologic treatment option for the prevention of thrombotic stroke in patients with nonvalvular atrial fibrillation (AF) who have contraindications to long‐term oral anticoagulation.^1^ Several clinical trials and observational studies have established the safety and clinical effectiveness of the first‐generation WATCHMAN LAAC device for reducing the risk of AF‐related embolic strokes in patients at high risk.^2^ This first‐generation WATCHMAN device has been evaluated in multiple clinical studies and has been implanted in >100 000 patients worldwide. The next‐generation WATCHMAN FLX device (Boston Scientific, Marlborough, MA) was designed to address and improve certain limitations observed with the first‐generation device, including an incomplete size matrix, inability to fully recapture the device during implantation, risk of perforation, prevention of device embolization, and residual peridevice leak. The safety and effectiveness of this next‐generation LAAC device was evaluated in the PINNACLE FLX US Food and Drug Administration Investigational Device Exemption clinical trial. The 1‐year primary outcome data have been published.^3^ The primary safety end point of 7 days/discharge adverse event rate was 0.5% (2/400) with a 2‐sided 95% CI of 1.6, which was significantly (*P*<0.0001) below the prespecified performance goal of 4.21%. The primary effectiveness end point of effective LAAC at 1 year was 100% (n=342), with a lower 1‐sided 95% CI of 99.1%, which was significantly above the prespecified performance goal of 97.0% (*P*<0.0001). At 1 year, 90% of patients had no detectable peridevice leak and, among those with any degree of residual peridevice leak, all jet sizes were measured as ≤3 mm.^3^

These final 2‐year results of the trial represent the first report of longer‐term outcomes with this next‐generation LAAC device.

## METHODS

The PINNACLE FLX (Protection Against Embolism for Non‐valvular AF Patients: Investigational Device Evaluation of the Watchman FLX LAA [Left Atrial Appendage] Closure Technology) trial is a single‐arm, prospective, nonrandomized trial designed to evaluate the safety and performance of this next‐generation WATCHMAN FLX LAAC device. Materials and methods have been previously described in detail^3^ and are briefly summarized herein and in Data S1. The data supporting this publication may be made available to other researchers in accordance with Boston Scientific's Data Sharing Policy (available at https://www.bostonscientific.com/en‐US/data‐sharing‐requests.html).

A total of 400 patients were enrolled across 29 investigational centers in the United States. Patients were eligible for enrollment if they had nonvalvular AF and a CHA_2_DS_2_‐VASc score of ≥2 for men or ≥3 for women, were able to take the prescribed postimplant antithrombotic medication regimen, had a rationale for a nonpharmacologic approach to stroke prevention, and had no other diagnoses that would require long‐term anticoagulation. Institutional Review Board approval was obtained at all sites, and all patients gave written informed consent before enrollment.

Adverse events were adjudicated by an independent clinical events committee (CEC), and imaging was independently assessed by a core laboratory (MedStar Health Research Institute; Data S1). Bleeding events were categorized by the CEC according to the Bleeding Academic Research Consortium definitions.

### Procedure and Follow‐Up

Procedural details for LAAC device implantation have been previously described^3^ and are provided in Data S1.

### Outcomes Measures

Primary end points have been previously described^3^ and are provided in Data S1. The prespecified secondary effectiveness end point was the occurrence of ischemic stroke or systemic embolism at 2 years.

### Statistical Analysis

For the secondary effectiveness end point of 2‐year CEC‐adjudicated ischemic stroke or systemic embolism, a performance goal of 8.7% was established. This value was based on an expected rate of 4.7%, as observed in PREVAIL (Prospective Randomized Evaluation of the WATCHMAN LAA Closure Device in Patients with Atrial Fibrillation versus Long Term Warfarin Therapy)‐eligible subjects with a CHADS_2_ score ≥2 or a CHA_2_DS_2_‐VASc score ≥3 from the combined LAAC device arms of the PROTECT‐AF (Watchman Left Atrial Appendage System for Embolic Protection in Patients With Atrial Fibrillation), CAP (Continued Access to PROTECT‐AF Registry), PREVAIL, and CAP2 (Continued Access to PREVAIL AF Registry) studies.^2^, ^4^, ^5^ A Δ of 4.0% was added to the expected event rate to establish the performance goal of 8.7%. This Δ was chosen on the basis of variability in event rates in the prior clinical studies and was considered clinically reasonable. Additional information on sample size calculation and analysis is provided in Data S1.

## RESULTS

### Study Population and Procedural Characteristics

Baseline demographics and procedural characteristics have been previously published,^3^ with selected characteristics shown in Table S1. Briefly, among 400 enrolled patients, the mean age was 73.8±8.6 years (86% were aged ≥65 years), 35.5% of the patients were women, and 94% were White race, with 4.7% Black race, 2.6% Hispanic or Latino ethnicity, and 0.8% Asian or American Indian or Alaska Native race. Approximately half of the patients presented with nonparoxysmal AF. The mean baseline CHA_2_DS_2_‐VASc score was 4.2±1.5, and the mean HAS‐BLED score was 2.0±1.0.

The next‐generation LAAC device was successfully implanted in 395 (98.8%) patients, defined as the successful delivery and release of the LAAC device in the correct position in the LAA. In the 5 patients who did not have successful implantation, 3 were attributable to unsuitable anatomy (eg, excessive vascular tortuosity or insufficient device anchoring), and 2 were attributable to inadequate final device compression and/or LAA seal. The most common device size implanted was 27 mm (31.1%); 11.4% of patients received a 20‐mm device, and 7.8% received a 35‐mm device; note that neither of these extreme sizes (20 and 35 mm) was previously available with the first‐generation device (Table S1).

### Outcomes at 2 Years

#### Clinical Follow‐Up

Two‐year clinical follow‐up was completed for 96.9% (373 of 385) patients. A total of 15 patients withdrew by 2 years: (1) 5 patients who were not successfully implanted were not followed up beyond 45 days per protocol, and (2) 10 additional patients withdrew consent. An additional 12 patients missed the 2‐year clinical visit with no subsequent clinical follow‐up.

#### Medications

Medication use at 1 and 2 years is shown in the Table. Per protocol, most implanted patients received a non–vitamin K oral anticoagulant (OAC; 76.7% apixaban, 20.3% rivaroxaban, 2.0% dabigatran, and 0.3% edoxaban) and aspirin after discharge through 45 days. By 12 months, <10% of patients each were still taking an OAC or P2Y12 inhibitor, whereas 97% continued taking aspirin. At 2 years, 7% of patients were taking an OAC, 6.2% were receiving dual‐antiplatelet therapy, and 96% were taking aspirin. Through 2 years, 58 patients resumed OAC, most commonly for cardiac ablation or cardioversion (39.7% [23/58]), followed by deep vein thrombosis/pulmonary embolism treatment or prophylaxis (24.1% [14/58]), and device related thrombus (10.3% [6/58]), as previously reported.^3^

**Table .** Clinical Outcomes

Outcome	Value at 1 y	Value at 2 y
Medication use*		
Oral anticoagulation	6.5	7.1
Dual‐antiplatelet therapy	5.9	6.2
Aspirin alone	96.9	95.3
Safety†		
All‐cause mortality	6.4 (25)	9.3 (36)
Cardiovascular or unknown mortality	4.2 (16)	5.5 (21)
All stroke	2.9 (11)	3.4 (13)
Ischemic stroke	2.9 (11)	3.1 (12)
Hemorrhagic stroke	0 (0)	0.3 (1)
Systemic embolism	0.3 (1)	0.3 (1)
Major bleeding	8.2 (32)	10.1 (39)
BARC 3	7.7 (30)	9.6 (37)
BARC 5	0.5 (2)	0.5 (2)
Pericardial effusion requiring intervention‡	1.0 (4)	1.0 (4)
Requiring open cardiac surgery	0 (0)	0 (0)
Requiring pericardiocentesis or pericardial puncture	1.0 (4)	1.0 (4)
Device embolization	0 (0)	0 (0)

BARC indicates Bleeding Academic Research Consortium.

* Values are percentage of patients who reported being on that medication at the time of visit. Patients may have been on >1 medication, so values may not total to 100%.

^†^ Values are Kaplan‐Meier cumulative estimates of clinical events committee–adjudicated outcomes, expressed as percentage (number).

^‡^ Pericardial effusion requiring either open surgery or pericardiocentesis.

#### Secondary Efficacy End Point

At 2 years, the CEC‐adjudicated rate of ischemic stroke or systemic embolism was 3.4% (annualized rate, 1.7%), with a 95% upper 1‐sided confidence bound of 5.3%. This was below the performance goal of 8.7%; therefore, the end point was met (Figure [A]). The rate at 1 year was 3.1% and, as shown in the Kaplan‐Meier curve (Figure [B]), only 1 patient had a new additional ischemic event after 1 year.

**Figure .**
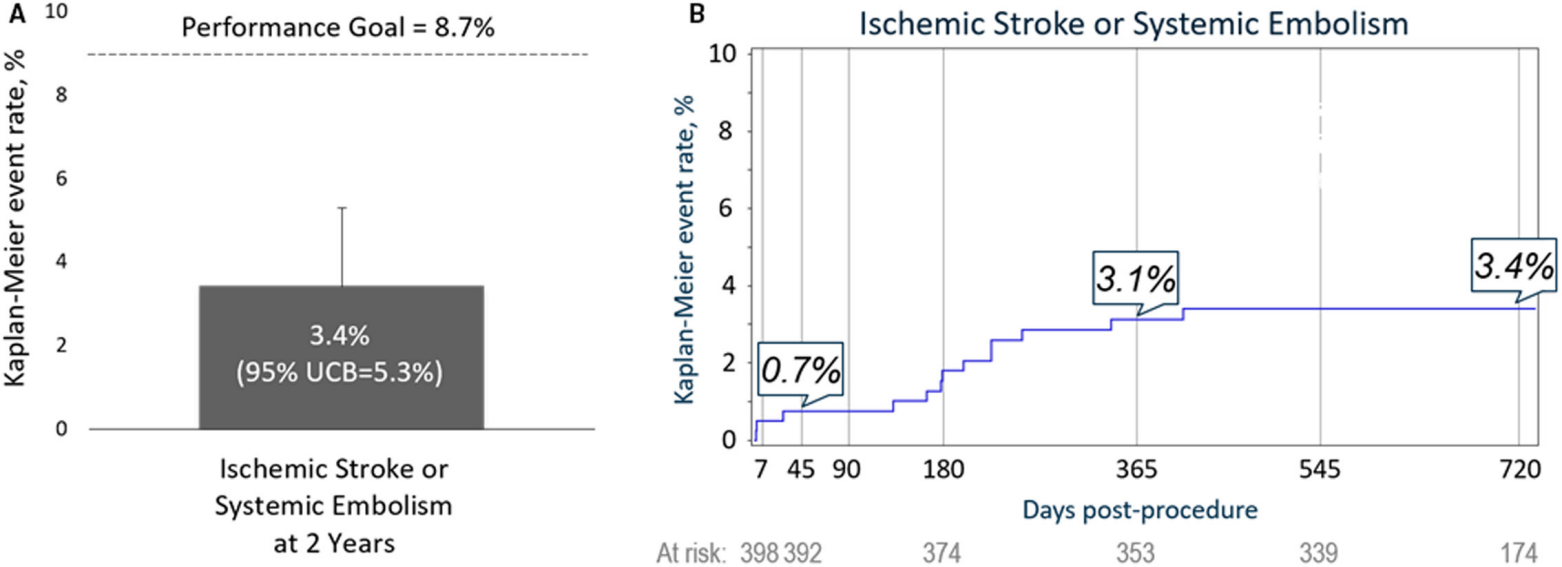
PINNACLE FLX (Protection Against Embolism for Non‐valvular AF [Atrial Fibrillation] Patients: Investigational Device Evaluation of the Watchman FLX LAA [Left Atrial Appendage] Closure Technology) secondary efficacy end point analysis. **A**, The prespecified secondary efficacy end point was met with a rate of 3.4% at 2 years and an upper 1‐sided 95% confidence bound (UCB) of 5.3%, which was below the performance goal of 8.7%. **B**, Kaplan‐Meier estimates of the rate of ischemic stroke or systemic embolism through 2 years.

#### Other Clinical Outcomes at 2 Years

Clinical outcomes at 1 and 2 years are shown in the Table. The 2‐year rate of all‐cause mortality was 9.3%, with approximately half of all new deaths between 1 and 2 years from noncardiovascular causes. As noted above, 1 patient experienced a new ischemic stroke after 1 year, and 1 patient experienced a hemorrhagic stroke between 1 and 2 years. The patient who experienced the hemorrhagic stroke was taking apixaban and aspirin at the time of the event, having previously experienced a device‐related thrombus/systemic embolism at day 175 after procedure while taking dual‐antiplatelet therapy. More than 70% of all Bleeding Academic Research Consortium 3 or 5 bleeding events occurred within 6 months of procedure, when patients were protocol mandated to be on OAC or dual‐antiplatelet therapy. Between 1 and 2 years, 1.9% of patients experienced major bleeding, all of which were CEC classified as Bleeding Academic Research Consortium 3. No device‐related thrombus or pericardial effusion requiring intervention (open surgery or pericardiocentesis) was reported after 1 year. No patient experienced a device embolization throughout the entire 2 years of the study.

## DISCUSSION

The next‐generation WATCHMAN FLX was designed to address several limitations of the predicate device, including reduced pericardial effusions, improved LAA sealing, and enhanced device stability. The primary results of the PINNACLE FLX trial demonstrated excellent procedural safety and substantially lower peridevice leak compared with prior studies. Whether these early findings translate into improved longer‐term clinical outcomes has not been evaluated.

These final results of the PINNACLE FLX US approval trial demonstrate the sustained safety and efficacy of the next‐generation LAAC device through 2 years. The prespecified secondary end point of long‐term ischemic stroke/systemic embolism was met, with a rate of 3.4% (annualized rate, 1.7%) and an upper 95% confidence bound of 5.3%, which was better than the performance goal of 8.7%. In longer‐term outcomes, between 1 and 2 years, 1 additional patient experienced an ischemic event, and no additional device related thrombus or pericardial effusions requiring intervention were reported. No device embolizations were reported throughout the entire study.

As published previously, similarly low ischemic stroke rates per 100 patient years were noted after LAAC with the legacy first‐generation LAAC device.^2^, ^4^, ^5^, ^6^, ^7^, ^8^, ^9^ Moreover, reduction in overall stroke rates versus warfarin in patients with nonvalvular AF has been clearly demonstrated in multiple trials of direct OAC therapy, which reported annualized overall stroke rates of 1.0% to 1.9% per year at ≈2 years of follow‐up^10^, ^11^, ^12^, ^13^; this compares well with the annualized overall stroke rate of 1.7% per year at 2 years in this study. Similarly, the 2‐year mortality rate observed in this study is also consistent with other studies of patients with nonvalvular AF with similar baseline CHA_2_DS_2_VASc scores. Two‐year all‐cause mortality rates in other device arms of LAAC studies with baseline CHA_2_DS_2_VASc score of 4.0 to 4.5 have ranged from 7.6% to 16.4%,^5^, ^7^, ^9^, ^14^ in comparison to the 9.4% observed in PINNACLE FLX trial.

Prior studies suggest that peridevice leak may be associated with ischemic stroke.^15^ The rate of complete anatomic LAAC with the next‐generation LAAC was 90%, and all residual leaks were <3 mm at 1‐year follow‐up. This closure rate was substantially higher than that observed in previous reports of the first‐generation device,^3^ likely a result of improvements in device design and implantation technique.

The infrequent occurrence of ischemic stroke beyond 1 year, only 1 additional patient had an ischemic stroke event between year 1 and 2, may be related to the enhanced rate of anatomic closure, although this remains speculative, as the study design was observational and the sample size relatively small to robustly define the rates of ischemic stroke and systemic embolism. This remains to be conclusively proven.

When comparing OACs, with the exception of dabigatran, ischemic stroke rates were similar between the other direct OACs and warfarin. The safety and long‐term clinical efficacy of the WATCHMAN FLX device compared with direct OACs in a contemporary population of OAC‐tolerant patients is underway and will be established by the WATCHMAN FLX Versus NOAC for Embolic ProtectION in the Management of Patients With Non‐Valvular Atrial Fibrillation (CHAMPION‐AF) randomized trial (ClinicalTrials.gov Identifier: NCT04394546).

### Study Limitations

Our study has several limitations. First, rather than a randomized trial, this was a single‐arm study compared with a performance goal based on results with the predicate first‐generation LAAC device. Second, because patients were required to take oral anticoagulation until demonstration of LAA seal, our results may not be generalizable to patients who have absolute contraindications to OAC therapy. Third, the 4% Δ for the secondary efficacy end point contains an element of arbitrariness, although it was chosen after consideration of observed variability in prior studies and was deemed to be clinically reasonable. Finally, the study sample size may not be large enough to provide robust estimates of the rates of rare clinical events.

## CONCLUSIONS

In the PINNACLE FLX trial, the next‐generation WATCHMAN FLX device demonstrated 100% effective closure of the LAA and low rates of safety events at 2 years. The prespecified, powered key secondary end point of stroke or systemic embolism was successfully met with a low rate of additional late stroke.

## APPENDIX

### The PINNACLE FLX Investigators

Stuart Adler, M Health Fairview East Region, St Paul, MN; Maurice Buchbinder, Sharpe Chula Vista Medical Center, San Diego, CA; Larry Chinitz, New York University Medical Center, New York, NY; David Delurgio, Emory University Hospital, Atlanta, GA; Amish Desai, Legacy Emanuel Hospital and Health Center, Portland, OR; Shephal Doshi, Saint John's Health Center, Pacific Heart Institute, Santa Monica, CA; Srinivas R. Dukkipatti, Helmsley Electrophysiology Center, Icahn School of Medicine at Mount Sinai, New York, NY; Christopher Ellis, Vanderbilt University Medical Center, Nashville, TN; Douglas Gibson, Scripps Memorial Hospital, La Jolla, CA; David Holmes, Mayo Clinic Foundation, Rochester, MN; Rodney Horton, Texas Cardiac Arrhythmia Research, Austin, TX; Kenneth Huber, St. Luke's Hospital of Kansas City, Kansas City, MO; Saibal Kar, Los Robles Regional Medical Center, Thousand Oaks, CA; Farhat Khairallah, Tallahassee Memorial Hospital, Tallahassee, FL; Jamie Kim, New England Heart and Vascular Institute at Catholic Medical Center, Manchester, NH; Jayanthi Koneru, Virginia Commonwealth University Health System, Richmond, VA; Paul Mahoney, Sentara Norfolk General Hospital, Norfolk, VA; Moussa Mansour, Massachusetts General Hospital, Boston, MA; George Mark, Cardiovascular Associates of the Delaware Valley, Sewell, NJ; Devi Nair, St. Bernard's Medical Center, Jonesboro, AR; William O'Neill, Henry Ford Hospital, Detroit, MI; William Nicholson, York Hospital, York, PA; Jose Osorio, Arrhythmia Institute at Grandview, Birmingham, AL; Matthew J. Price, Scripps Clinic, La Jolla, CA; Vivek Y. Reddy, Helmsley Electrophysiology Center, Icahn School of Medicine at Mount Sinai, New York, NY; James Reiss, PeaceHealth Southwest Medical, Vancouver, WA; Ashish Sadhu, Phoenix Cardiovascular Research Group, Phoenix, AZ; Walid Saliba, Cleveland Clinic Foundation, Cleveland, OH; Manish Shah, MedStar Health Medical Center, Washington, DC; Michael Shehata, Cedars‐Sinai Medical Center, Los Angeles, CA; James Stone, Jr, North Mississippi Medical Center, Tupelo, MS; Vijendra Swarup, Arizona Arrhythmia Research Group, Phoenix, AZ.

## Sources of Funding

The PINNACLE FLX (Protection Against Embolism for Non‐valvular AF [Atrial Fibrillation] Patients: Investigational Device Evaluation of the Watchman FLX LAA [Left Atrial Appendage] Closure Technology) trial was sponsored and funded by Boston Scientific, Inc (Marlborough, MA).

## Disclosures

Shephal K. Doshi is a consultant to Boston Scientific, Biosense Webster, Abbott Vascular, and Conformal Medical; and is coprincipal investigator of the PINNACLE FLX, CHAMPION AF, and Conform IDE trials. Saibal Kar is a consultant to Boston Scientific, Abbott Vascular, Medtronic, and Laminar; and is coprincipal investigator of the PINNACLE FLX and CHAMPION AF trials. Ashish Sadhu reports consulting honoraria from Boston Scientific. Rodney Horton reports advisory board participation with Boston Scientific, Biosense Webster, and Abbott; and consulting honoraria from Boston Scientific. Jose Osorio reports Boston Scientific advisory board participation; consulting honoraria from Boston Scientific, Biosense‐Webster, Medtronic (institution), and Galaxy Medical; and research grants from Boston Scientific, Biosense‐Webster, and Medtronic (institution). Christopher Ellis reports research funding paid to Vanderbilt Medical Center from Medtronic, Atricure, Boehringer Ingelheim, and Boston Scientific; and consulting honoraria from Abbott Vascular, Atricure, Boston Scientific, and Medtronic. James Stone, Jr, reports reimbursement for proctoring and physician training from Boston Scientific; and funding for clinical research from Boston Scientific, Johnson & Johnson, and Abbott Vascular. Manish Shah reports consulting honoraria from Boston Scientific. Srinivas R. Dukkipati reports a research grant from Biosense Webster; and equity in Farapluse and Manual Surgical Sciences. Stuart Adler reports consulting honoraria from Medtronic. Devi G. Nair reports consulting honoraria and research grants from Boston Scientific, Abbott, Johnson & Johnson, and Medtronic. Jamie Kim reports consulting and speaker's honoraria from Boston Scientific, Conformal Medical, and Janssen. Oussama Wazni reports consulting and speaker's honoraria from Boston Scientific and Biosense Webster; and is principal investigator of the OPTION trial. Matthew J. Price reports consulting and speaker's honoraria from Astra‐Zeneca, Abbott Vascular, Boston Scientific, Chiesi USA, W.L. Gore Medical, and Medtronic; and research grants (to institution) from Daiichi Sankyo. Robert Shipley reports salary and equity (Boston Scientific). Thomas Christen reports salary and equity (Boston Scientific). Dominic J. Allocco reports salary and equity (Boston Scientific). Vivek Y. Reddy is an unpaid consultant to Boston Scientific; disclosures with medical companies include: Abbott, Axon, Biosense‐Webster, Biotronik, Boston Scientific, Cardiofocus, Cardionomic, CardioNXT/AFTx, EBR, Impulse Dynamics, Medtronic, Philips, Stimda, and Thermedical (consultant); Ablacon, Acutus Medical, Affera, Apama Medical, Aquaheart, Autonomix, Backbeat, BioSig, Circa Scientific, Corvia Medical, East End Medical, EPD, Epix Therapeutics, EpiEP, Eximo, Farapulse, Fire1, Javelin, Keystone Heart, LuxCath, Medlumics, Middlepeak, Nuvera, Valcare, and VytronUS (consultant, equity); and Manual Surgical Sciences, Newpace, Surecor, and Vizara (equity). David R. Holmes, Jr, has no disclosures to report.

## Supporting information

Data S1
Table S1
