# Supplementary material for: Two‐Year Outcomes With a Next‐Generation Left Atrial Appendage Device: Final Results of the PINNACLE FLX Trial
Source: J Am Heart Assoc. 2023 Feb 15;12(4):e026295. doi: 10.1161/JAHA.122.026295 (PMC10111496; doi:10.1161/JAHA.122.026295)
Supplement: Supplementary file 1 — Data S1 Table S1 [file JAH3-12-e026295-s001.pdf]

Supplemental Material

Data S1.

Supplemental Methods

Study Design and Conduct

This study was conducted in accordance with the International Conference on Harmonization Guidelines for Good Clinical Practice, the ethical principles outlined in the Declaration of Helsinki, and all applicable local/national regulations. The protocol was approved by the institutional review boards/ethics committees at each site prior to enrollment. The study was sponsored by Boston Scientific Corporation and registered with ClinicalTrials.gov (NCT02702271). The Principal Investigators of the PINNACLE FLX trial (Shephal Doshi and Saibal Kar) had full access to all of the data in the study and take responsibility for its integrity and the data analysis.

Procedure and Follow-up

Post-implant clinical follow-up visits were required at 7 days/hospital discharge (whichever came first), 45 days, and 6, 12, 18, and 24 months. Transesophageal echocardiography assessment was required post-procedure, at 45 days, again at 6 months if no effective LAA seal (defined as peri-device leak >5mm) was observed at 45 days, and at 1 year. Additional imaging was performed per physician discretion as clinically indicated (ie, in case of an adverse event).

Per protocol, post-procedure medication regimen consisted of treatment with a direct oral anticoagulant (DOAC), preferably apixaban or rivaroxaban, along with low-dose (81-100mg/day) aspirin through at least 45 days follow-up. Upon evidence of adequate LAA seal,

defined as leak ≤5mm, at the 45-day transesophageal echocardiography (TEE) evaluation, patients were directed to discontinue DOAC therapy and begin a dual anti-platelet therapy (DAPT) regimen of P2Y12 inhibitor, preferably clopidogrel (75mg), plus low dose aspirin until 6 months post-implant, followed thereafter by low dose aspirin indefinitely. If the 45-day TEE assessment showed a leak >5 mm, patients continued DOAC plus aspirin and were re-evaluated at 6-months post implant. If there were no leaks >5 mm at the subsequent follow up visit, patients could forego DAPT and proceed straight to low dose aspirin indefinitely. This recommended medication regimen could be changed at any time if, according to the judgement of the treating physician, it was in the best medical interest of the patient.

The primary safety endpoint was the occurrence of one of the following events between the time of implant and within seven days following the procedure or by hospital discharge, whichever was later: death, ischemic stroke, systemic embolism, or device- or procedure-related events requiring open cardiac surgery or major endovascular intervention such as pseudoaneurysm repair, arteriovenous fistula repair, or other major endovascular repair. Percutaneous catheter drainage of pericardial effusions, snaring of an embolized device, thrombin injection to treat femoral pseudoaneurysm and nonsurgical treatments of access site complications were excluded from this endpoint. The primary effectiveness endpoint was the rate of effective LAA closure, defined as any peri-device flow with jet size ≤5mm per core laboratory-assessed TEE at 12 months.

Statistical Methods

A sample size of 400 enrolled subjects was required to evaluate the secondary effectiveness endpoint, accounting for 20% attrition. This sample size was calculated employing exact binomial methodology and confirmed via Monte Carlo simulations employing Kaplan-Meier

methodology, using SAS version 9.4 with the following assumptions:

Expected event rate = 4.7%

Delta = 4.0%

Performance goal = 8.7%

1-sided alpha = 5%

Power = 90%

Expected attrition rate = 20%

Required sample size = 400 subjects

Data from all implanted or attempted subjects at the time of endpoint analysis were included. The 95% one-sided upper pointwise confidence limit of the event rate was calculated via log-log methodology for all eligible subjects contributing to the analyses and compared to the performance goal of 8.7%.

Baseline and procedural characteristics are summarized as means ± standard deviations for continuous variables and percent (n/N) for categorical variables. One- and two-year clinical outcomes rates were assessed using Kaplan-Meier estimates, with event-free subjects who exited the study censored at the time of last available follow-up. All statistical analyses were performed using SAS® version 9.4 or greater (Cary, NC, USA).

Table S1. Selected Baseline and Procedural Characteristics.

Variable	All Enrolled Subjects (N=400)
Age, years	73.8±8.6 (400)
Age ≥ 65 years	86.3% (345/400)
Female sex	35.5% (142/400)
Atrial fibrillation pattern	
Paroxysmal AF	51.8% (207/400)
Persistent AF	36.5% (146/400)
Permanent AF	10.5% (42/400)
Paced AF	1.3% (5/400)
CHA_2DS_2-VASc score	4.2 ± 1.5 (400)
HAS-BLED score	2.0±1.0 (400)
Diabetes	30.5% (122/400)
Prior stroke, transient ischemic event, or thromboembolism	22.3% (89/400)
Vascular disease	55.3% (221/400)
LVEF, %	56.1±8.4 (400)
LAA ostium diameter, mm	21.1±3.7 (400)
LAA length, mm	28.3±5.7 (400)
Multilobular LAA	35.0% (140/400)
Successful device implantation	98.8% (395/400)
Implanted device size	
20mm	11.4% (45/395)
24mm	26.8% (106/395)
27mm	31.1% (123/395)

31mm	22.8% (90/395)
35mm	7.8% (31/395)
>1 WATCHMAN device attempted	15.8% (63/400)
Partial device recaptures per implanted/attempted devices	1.8±2.8 (400)
Full device recaptures per implanted/attempted devices	0.4±1.1 (400)

Values are mean±standard deviation (n) or percent (n/N).
AF=atrial fibrillation; CHA_2DS_2-VASc = congestive heart failure, hypertension, 75 years of age and older, presence of diabetes, previous stroke or transient ischemic attack, vascular disease, 65 to 74 years of age, female sex; HAS-BLED =hypertension, abnormal renal/liver function, prior stroke, bleeding history or predisposition to bleeding, labile international normalized ratio, age ≥65 years, alcohol use >8 drinks per week. LAA=left atrial appendage; LVEF=left ventricular ejection fraction.
